# Association between the response of intravitreal antivascular endothelial growth factor injection and systemic factors of diabetic macular edema

**DOI:** 10.1186/s12886-024-03432-7

**Published:** 2024-04-15

**Authors:** So Hyung Lee, Geun Woo Lee, Soo Jung Lee, Seong Gyu Kim

**Affiliations:** 1 Department of Ophthalmology, Daegu Catholic University School of Medicine, Daegu, Republic of Korea; 2 Division of Nephrology, Department of Internal Medicine, Daegu Catholic University School of Medicine, #33 Duryugongwon-ro 17-gil, Nam-gu, 42472 Daegu, Republic of Korea

**Keywords:** Diabetic retinopathy, Glomerular filtration rate, Glycated hemoglobin, Macular edema, Intravitreal injection

## Abstract

**Background:**

This study investigated the effects of systemic factors in response to intravitreal injections in patients with macular edema due to non-proliferative diabetic retinopathy (NPDR).

**Methods:**

We retrospectively reviewed the medical records of patients treated with intravitreal injections for macular edema secondary to NPDR between January 2018 and January 2021. The patients were divided into three groups according to the injection response. When patients with diabetic macular edema showed 20µ or more reduction in central retinal thickness compared to baseline, they were classified as responsive group, and if not, they were classified as refractory group. The responsive group was further divided into the complete and incomplete response groups. Patients with complete disappearance of edema at seven months were classified as the complete response group, whereas those in which edema did not disappear were classified as the incomplete response group. The clinical characteristics of each group, including medical history, ophthalmic examination results, and laboratory examination results at the time of diagnosis, were analyzed.

**Results:**

Of the 112 eyes (91 patients) that satisfied the inclusion criteria, 89 (77 patients) in the responsive group and 23 (14 patients) in the refractory group were included in the analysis. The responsive group was further divided into the complete (51 eyes) and incomplete (38 eyes) response groups. The refractory group had significantly higher glycated hemoglobin levels and significantly lower estimated glomerular filtration rates than the responsive group (*p* = 0.026 and *p* = 0.012, respectively). In the multivariate logistic regression analysis, both factors were found to be significant in predicting the degree of response (all *p* < 0.05). No factor showed a significant difference between the incomplete and complete response groups(all *p* > 0.05).

**Conclusions:**

In macular edema caused by NPDR, low glomerular filtration rates and high glycated hemoglobin levels may be used as predictors of poor response to intravitreal injection therapy. In addition to blood glucose control, education should be provided regarding the need for the continuous monitoring of renal function.

## Background

Diabetic retinopathy is the most common cause of vision loss in patients with diabetes > 20 years. In chronic hyperglycemia, microvascular abnormalities and obstructions cause diabetic macular edema, which is the main cause of permanent vision loss during active age [1, 2]. 

The duration of diabetes and high glycated hemoglobin levels have been reported as factors associated with diabetic macular edema in patients with diabetic retinopathy [3, 4]. Furthermore, there have been numerous reports on the association between chronic kidney disease and the development and progression of diabetic retinopathy and macular edema [5, 6]. 

Diabetic macular edema can appear regardless of the severity of diabetic retinopathy, and if left untreated, it can cause serious visual impairment. Previously, focal laser therapy was administered; however, intravitreal injections of various drugs are the major treatments for diabetic macular edema [7]. Anti-vascular endothelial growth factor (anti-VEGF) and steroids are representative drugs. Due to the possible side effects of steroids, including cataracts, endophthalmitis, and increased intraocular pressure, intravitreal anti-VEGF injection is usually the first treatment [8, 9]. Anti-VEGF drugs such as bevacizumab, aflibercept, and ranibizumab are currently in use, as are steroids such as triamcinolone acetonide and dexamethasone implants, all of which are effective in the treatment of macular edema [10–12]. 

Numerous studies have investigated systemic factors associated with functional changes—such as visual acuity—and anatomical changes—such as central macular thickness, foveal avascular zone size, and posterior pole vessel density—which are measured using optical coherence tomography (OCT) and optical coherence tomography angiography (OCT-A) [13–15]. 

However, no study has identified the systemic factors associated with the degree of response to intravitreal anti-VEGF injections in diabetic macular edema. Therefore, we investigated the systemic factors associated with response to intravitreal anti-VEGF injections using OCT in patients first diagnosed with macular edema due to NPDR.

## Methods

This study adhered to the tenets of the Declaration of Helsinki and was approved by the Institutional Review Board of Daegu Catholic University Medical Center.

Patients with type 2 diabetes who visited the ophthalmology, endocrinology, or nephrology departments of Daegu Catholic University Hospital between January 2018 and January 2021 were included in this retrospective analysis. Eligible patients were initially diagnosed with macular edema due to NPDR and treated with intravitreal injections. Patients who received therapy and follow-up at an ophthalmology clinic for at least 7 months following their initial diabetic macular edema diagnosis were included. All the participants had blood and urine test results 2 months before or after the date of diagnosis. When two test results were available within the study period, the test closest to the date of diagnosis was evaluated. The following cases were excluded from the study:1) other retinal disorders that can cause macular edema, 2) cataract surgery within 6 months of diagnosis, 3) a history of intravitreal anti-VEGF injection treatment in either the observation or contralateral eye, 4) a history of vitrectomy or photocoagulation, 5) dialysis, and 6) a shift in diagnosis to proliferative diabetic retinopathy during treatment.

At the time of diagnosis and at each outpatient visit, all patients underwent bilateral best-corrected visual acuity, tonometry, mydriatic fundus examination, wide-angle fundus photography, and OCT (AngioVue®, Optovue, Fremont, California, USA). Corrected visual acuity was measured using the Snellen visual acuity chart, and central retinal thickness was defined as the average thickness of the innermost 1 mm circle of the Early Treatment Diabetic Retinopathy Study circle, which was automatically measured by the software in the OCT equipment.

**Fig. 1:**
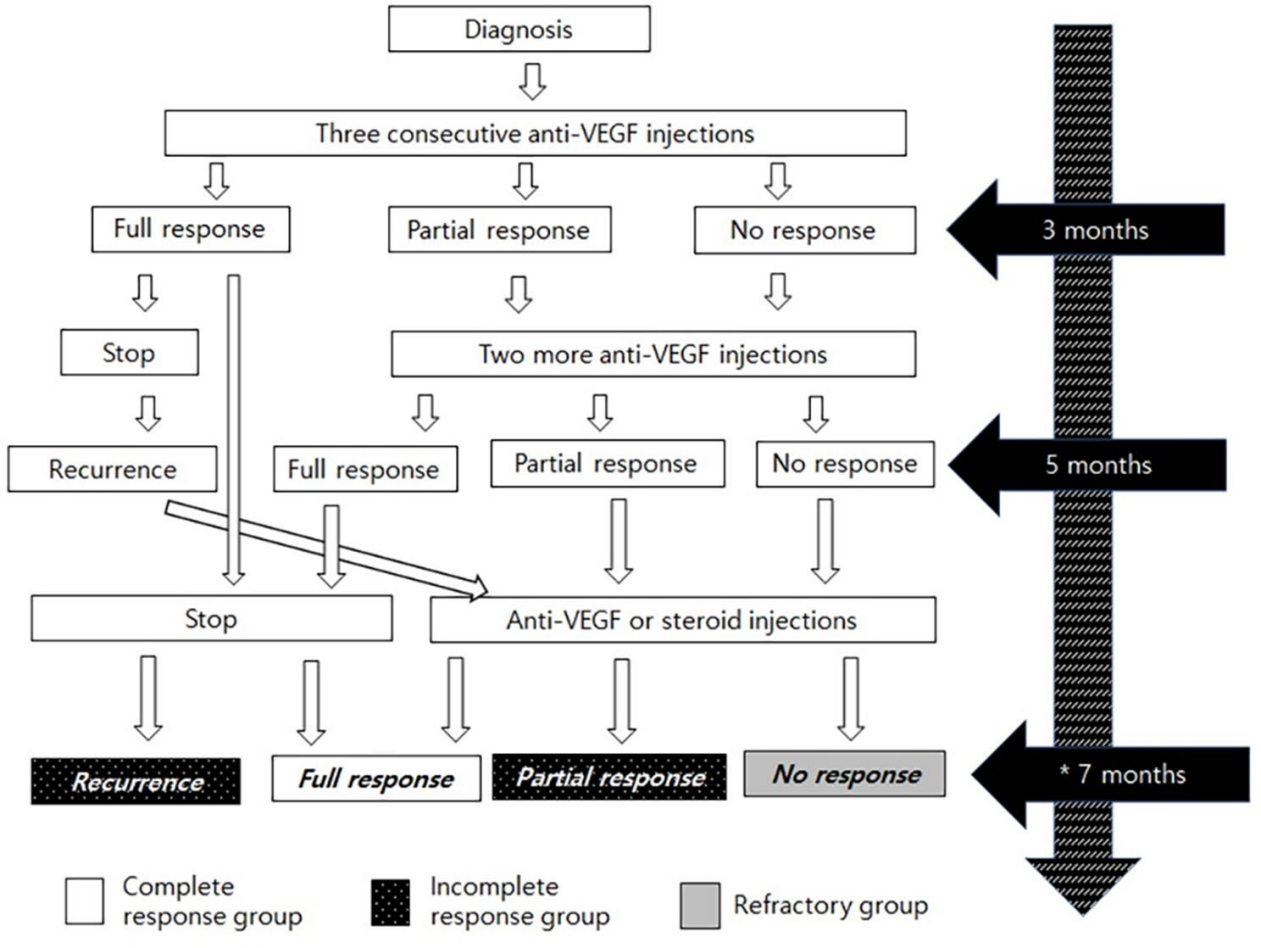
Timeline and classification of included participants. If a reduction in edema of at least 20 μm was confirmed through OCT at any time over 3, 5, and 7 months, it was included in the responsive group. If no such change was observed, the edema was included in the refractory group. The responsive group was divided into complete and incomplete response based on the OCT at 7 months. *Decision point of the group. VEGF = vascular endothelial growth factor. OCT = optical coherence tomography

After diagnosis, three consecutive intravitreal bevacizumab injections (Avastin®, Genentech, San Francisco, CA, USA) were administered. Treatment response three months after diagnosis determined whether only follow-up observation or two more bevacizumab injections would be necessary. In cases of insufficient response or recurrence 5 months after diagnosis, additional intravitreal bevacizumab or steroid injections were administered. The drug used for the steroid injection was dexamethasone (Ozurdex®, Allergan Inc., Irvine, CA, USA) or triamcinolone (Maqaid®, Hanmi Pharm Co, Seoul, Korea). The patients were then divided into two groups based on the OCT results at three and five months, as well as at the seventh month (decision point) after diagnosis When central retinal thickness decreases at least 20µ or more compared to baseline at any of the three time points, patients were defined as a responsive group; otherwise, they were labeled as a refractory group. Among the responsive group, those with complete disappearance of edema at seven months were classified as the complete response group, whereas those in which edema did not disappear were classified as the incomplete response group (Fig. 1).

Age, sex, duration of diabetes, history of hypertension, dyslipidemia, cerebrovascular or cardiovascular disease, and use of insulin were validated using the electronic records of each patient. The degree of NPDR was determined and classified according to severity at the time of diagnosis. The central retinal thickness and best-corrected visual acuity before injection were also used for analysis. Proteinuria, blood urea nitrogen, creatinine, glomerular filtration rate, glycated hemoglobin, and total cholesterol were analyzed as laboratory results.

The best-corrected visual acuity measured using the Snellen chart was converted to the logarithm of the minimum angle of resolution (logMAR) for statistical analysis. An independent t- or Mann-Whitney U-test was performed to compare the means of continuous variables between the two groups, and an X^2^ or Fisher’s exact test was used to compare the ratios. For factors with a p-value of 0.1 or less between the responsive group and the refractory group, multivariate logistic regression analysis was performed to investigate factors showing differences between the two groups. The Kruskal-Wallis test was performed to compare the refractory, complete response, and incomplete response groups. For significance, a post-hoc analysis was conducted to confirm the differences. Statistical analyses were performed using the SPSS statistics software package (version 21.0; IBM, Armonk, NY, USA), and a *p*-value less than 0.05 was considered significant.

## Results

A total of 91 patients were included in this study. Medical records and test data were analyzed for 77 participants (89 eyes) in the responsive group and 14 patients (23 eyes) in the refractory group.

Among the participants in the refractory group, the ratio of both eyes included was higher, with 12 patients in the responsive group and 9 patients in the refractory group (15.6% vs. 75.0%). The differences between the two groups are presented in Table 1.

**Table 1:** Demographics and clinical characteristics

Parameters	Total(*n* = 112)	Response group(*n* = 89)	Refractory group(*n* = 23)	*p*-value
Age, years	55.5 ± 10.2	55.2 ± 10.9	55.7 ± 10.8	*0.843
Sex, Male: Female	81:31:00	65:24:00	16:07	†0.657
DM duration, years	10.5 ± 7.6	9.3 ± 6.8	13.2 ± 8.1	‡0.063
severe NPDR, n(%)	91(81.3%)	70(78.6%)	21(91.3%)	†0.237
HTN, n(%)	83(74.1%)	66(74.2%)	17(73.9%)	†0.782
Hyperlipidemia, n(%)	72(64.2%)	60(67.4%)	12(52.2%)	†0.283
CVA history, n(%)	6(0.05%)	4(0.04%)	2(0.08%)	§ 0.212
CVD history, n(%)	7(0.06%)	5(0.05%)	2(0.08%)	§ 0.276
insulin using, n(%)	38(33.9%)	27(30.3%)	11(47.8%)	†0.102
PreCMT, µ	456.3 ± 71.8	450.2 ± 62.3	465 ± 73.3	‡0.113
PreBCVA, logMAR	0.52 ± 0.23	0.50 ± 0.22	0.54 ± 0.24	‡0.507
albuminuria, n(%)	36(32.1%)	23(25.8%)	13(56.5%)	†0.082
BUN, mg/dL	27.9 ± 11.1	26.7 ± 8.4	29.5 ± 13.3	‡0.121
Creatinine, mg/dL	2.8 ± 1.0	2.3 ± 1.2	3.5 ± 0.9	‡0.076
eGFR, mL/min/1.7m^2^	22.9 ± 11.8	26.8 ± 12.5	16.6 ± 10.6	‡0.012
HbA1c, %	7.45 ± 2.62	7.28 ± 2.34	7.88 ± 2.93	‡0.026
Total cholesterol, mg/dL	149.3 ± 36.5	147.4 ± 40.5	151.2 ± 28.7	*0.418

Continuous variables are reported as mean ± standard deviation values

DM = diabetes mellitus, NPDR = nonproliferative diabetic retinopathy, HTN = Hypertension, CVA = cerebrovascular accident, CVD = cardiovascular disease, CMT = central macular thickness, BCVA = Best corrected visual acuity, logMAR = logarithm of the minimum angle of resolution, BUN = blood urea nitrogen, eGFR = estimated glomerular filtration rate, HbA1c = hemoglobin A1c, *Independent t-test, †X square test, ‡Mann whitney U test, §Fisher’s exact test

The average age of all patients was 55.5 ± 10.2 years, and there was no difference between the two groups (*p* = 0.843). Additionally, there were no significant differences between the two groups in terms of sex, hypertension, dyslipidemia, severe NPDR, history of vascular disease, or insulin injection (all *p* > 0.05). There were no significant differences between the two groups in the best-corrected visual acuity, central retinal thickness, blood urea nitrogen, or total cholesterol levels before injection (all *p* > 0.05). Although the duration of diabetes, presence of proteinuria, and creatinine levels were higher in the refractory group, there were no significant differences between the two groups (*p* = 0.063, 0.082, and 0.076, respectively). However, glomerular filtration rate and glycated hemoglobin levels were significantly different between the two groups (*p* = 0.012 and 0.026, respectively).

Table 2 presents the results of the comparison between the complete and incomplete response groups. There were no significant differences between the two groups in factors such as duration of diabetes, presence of proteinuria, creatinine level, glomerular filtration rate, and glycated hemoglobin (all *p* > 0.05).

**Table 2:** Comparison between complete response and incomplete response

Parameters	Complete response(*n* = 51)	Incomplete response(*n* = 38)	*p*-value
DM duration, years	8.9 ± 6.6	9.5 ± 7.1	*0.511
insulin using, n(%)	15(29.4%)	12(31.6%)	†0.433
PreCMT, µ	452.6 ± 66.3	448.3 ± 61.1	*0.316
PreBCVA, logMAR	0.48 ± 0.21	0.52 ± 0.23	*0.273
albuminuria, n(%)	13(25.5%)	10(26.3%)	†0.631
BUN, mg/dL	24.7 ± 7.7	27.7 ± 9.1	*0.281
Creatinine, mg/dL	2.2 ± 0.9	2.4 ± 1.3	*0.236
eGFR, mL/min/1.7m^2^	27.8 ± 11.8	26.1 ± 12.8	*0.341
HbA1c, %	7.16 ± 2.31	7.30 ± 3.01	*0.221
Total cholesterol, mg/dL	145.5 ± 38.9	149.1 ± 41.8	*0.412

DM = diabetes mellitus, CMT = central macular thickness, BCVA = Best corrected visual acuity, logMAR = logarithm of the minimum angle of resolution, BUN = blood urea nitrogen, eGFR = estimated glomerular filtration rate, HbA1c = hemoglobin A1c, *Independent t-test, †X square test

To find factors associated to response, multivariate logistic regression analysis was performed using factors with a p-value of less than 0.1 (Table 3). Five factors were used in the analysis, and the expression—including only the glomerular filtration rate and glycated hemoglobin level—was a significant model (R^2^ = 0.501, goodness-of-fit test *p* = 0.226).

**Table 3:** *Multivariate logistic regression analysis for associated factors with response

Parameters	Adjusted OR	95% CI	*p*-value
DM duration, years			0.125
Albuminuria			0.183
Creatinine, mg/dL			0.094
†eGFR, mL/min/1.7m^2^	1.043	1.010–1.076	0.008
†HbA1c, %	1.035	1.007–1.064	0.01

*Model selected by multivariate logistic regression analysis with backward elimination(R^2^ = 0.501 and *p* = 0.226 by the Hosmer–Lemeshow test for goodness of fit, †selected parameters in model)

OR = odds ratio, CI = confidence interval, DM = diabetes mellitus, eGFR = estimated glomerular filtration rate, HbA1c = hemoglobin A1c

**Fig. 2:**
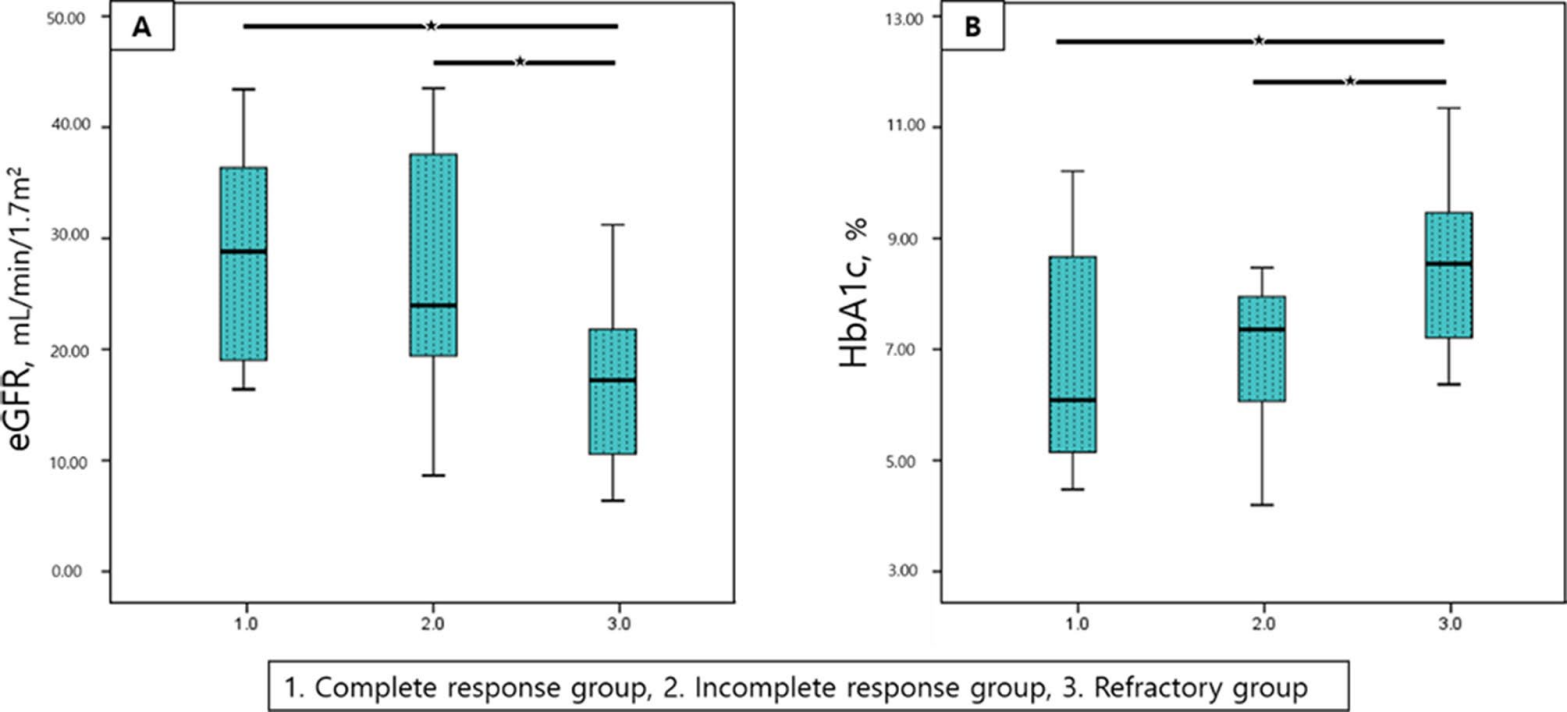
Box plots of two factors (eGFR, HbA1C) in three groups (error bar means 95% confidence interval). Kruskal Wallis analysis was performed on each of the factors (all *p* < 0.001). Posthoc analysis used the Mann Whitney *U* test, a *P* value < 0.01 was marked as an asterisk. eGFR = estimated glomerular filtration rate, HbA1c = hemoglobin A1c

Two factors, glomerular filtration rate and glycated hemoglobin, which showed significant differences between the responsive and refractory groups, were compared between the complete response, incomplete response, and refractory groups (Fig. 2). Both factors showed significant differences among the three groups (all *p* < 0.001). The post-hoc test showed that the refractory group had a significantly lower glomerular filtration rate and higher glycated hemoglobin level than the other two groups (all *p* < 0.01). But these two factors didn’t show significant difference between complete and incomplete groups.

## Discussion

Chronic hyperglycemia causes systemic microvascular abnormalities, particularly complications in the eyes and kidneys. In type 1 diabetes, an extended period of the disease often correlates with an increased frequency of diabetic retinopathy, a condition that commonly appears before diabetic nephropathy [16]. Uncontrolled diabetes also increases the risk of diabetic retinopathy and nephropathy in type 2 diabetes [17]. Thus, the two diseases are inevitably related in terms of diabetic complications. This study analyzed whether various systemic factors, including renal function, are related to the treatment response of macular edema due to diabetic retinopathy.

The patients were divided into responsive and refractory groups according to the degree of response to intravitreal bevacizumab injection. Factors related to the exacerbation of diabetic retinopathy, such as dyslipidemia, history of vascular disease, and use of insulin injections, best-corrected visual acuity, presence of severe NPDR, and central retinal thickness before injection did not show any difference according to the degree of response to the injection.

Diabetic retinopathy is a multifactorial disease that is related to systemic factors such as the duration of diabetes, glycated hemoglobin, cholesterol levels, and kidney function [5]. In this study, patients in the refractory group were more likely to have both eyes included than those in the responsive group. This indicates that the refractory group was more systemically affected by diabetes. In other words, it can be thought that systemic factors will deteriorate more in the refractory than in the responsive group and systemic factors will affect treatment response.

Diabetic nephropathy is often concurrent with diabetic retinopathy, and it is thought to be linked to the development of diabetic macular edema. Jeng et al. [18], however, suggests that diabetic nephropathy is related to the aggravation of diabetic retinopathy rather than the development of diabetic macular edema. However, Kume et al. [19] recently published a study on systemic factors associated with diabetic macular edema in the Japanese population which reported a significant relationship between diabetic nephropathy and diabetic macular edema. They explain that oxidative stress and decreased osmotic control ability following hyperglycemia deteriorates not only the kidney function but also the function of the blood-retinal barrier and retinal pigment epithelium of the eye. Hwang et al. [20] demonstrated that diabetic macular edema decreased after the first dialysis in patients with chronic nephropathy and that renal function may influence the development and treatment of diabetic macular edema. Other studies have found that anti-VEGF injection therapy is less effective in patients with high glycated hemoglobin levels, high blood creatinine levels, and a low glomerular filtration rate [14, 21]. In this study, the effect of the injection treatment decreased as renal function—indicated by the presence of proteinuria, blood urea nitrogen, and creatinine—decreased. However, the glycated hemoglobin levels and only the glomerular filtration rate among renal functions showed significant differences between the responsive and refractory groups in multivariate regression analysis. This means that the glucose control state has a more significant correlation with the treatment response of diabetic macular edema for intravitreal injection than the duration of diabetes. In addition, the glomerular filtration rate was selected as the more accurate parameter for kidney function, as compared to the presence of proteinuria and creatinine levels, which can be influenced by diet or other systemic conditions. The pathological similarities between diabetic retinopathy and chronic kidney disease may explain why the glycated hemoglobin levels and glomerular filtration rate correlate with the treatment response of diabetic macular edema for anti-VEGF injection. Endothelial cells in the retina, like mesenteric cells in the kidney, cannot regulate the concentration of glucose inside the cells in a hyperglycemic state, resulting in an increased secretion of inflammatory cytokines and VEGF, damaging the surrounding tissues [1]. Whereas, in this study, all the factors we analyzed including glomerular filtration rate and the glycated hemoglobin didn’t show significant difference between complete and incomplete groups. This suggest that there might be other factors affecting the degree of response of diabetic macular edema to intravitreal bevacizumab or steroid injection. Further studies with longer follow up period are needed to find these factors.

Although some studies have shown that anti-VEGF injection therapy is not associated with systemic factors [13–15, 18], differences in research results may occur because there is no common standard for comparing the effects of injection therapy and the patient groups recruited in each study are diverse. Particularly, in proliferative diabetic retinopathy, the degree of vascular damage varies, and it may be difficult to accurately determine the effect of injection treatment owing to hemorrhage or pan-retinal photocoagulation during intravitreal injection [21]. Therefore, patients with NPDR were chosen for this study, and the effect of intravitreal anti-VEGF injection therapy was measured based on changes in the central retinal thickness.

The clinical use of OCT-A has been on the rise because of its ability to detect the vascular structure of the macula noninvasively and efficiently. Research on diabetic retinopathy and diabetic macular edema using OCT-A is constantly being published [22–24], and more meaningful results can be obtained in future studies if the results of OCT-A are used for analysis.

This study has several limitations. First, it was conducted retrospectively on a small number of patients. Due to the nature of the retrospective study, we chose a follow-up period of at least 7 months since the period often varies after 7 months, related to treatment response. This study may be limited by varying decision points among ophthalmologists due to differences in treatment regimens. Second, the possibility of selection bias cannot be ruled out, because patients with NPDR from our hospital’s endocrinology or nephrology departments were selected. Furthermore, because the follow-up period was relatively short, studies on the long-term outcomes should be conducted. Another limitation is that after 5 months of treatment, the drugs used for intravitreal injection were not uniform. However, it is thought that these characteristics produced better results for reference in real-world clinical practice. Additionally, the criteria to classify response group and refractory group might be obscure. As there are no uniform criteria for treatment response of diabetic macular edema, we set the criteria considering our clinical experience, that patients with diabetic macular edema which doesn’t show minimal anatomical improvement usually had no treatment response eventually. Better results may have been obtained if the degree of disease control for hypertension and dyslipidemia had been assessed.

## Conclusions

In conclusion, low glomerular filtration rate and high glycated hemoglobin can be used as predictors of poor response to intravitreal injection therapy in macular edema due to NPDR. Using these test results, clinicians should explain patients with diabetes the treatment prognosis, the need for continuous monitoring of renal function as well as blood sugar management. Continuous intravitreal injection treatment for diabetic macular edema can be a financial burden due to its chronic nature. Given this, forewarning patients about poor treatment response of diabetic macular edema in the case of inadequate diabetes management or poor renal function can help increase patient compliance.

## Data Availability

Data used to support the findings of this study are available from the corresponding author upon request.

## Abbreviations

NPDR: non-proliferative diabetic Retinopathy
VEGF: vascular endothelial growth factor
OCT: Optical Coherence Tomography
OCT-A: optical coherence tomography angiography
logMAR: logarithm of the minimum angle of resolution
